# Structural and optical properties of amorphous Si–Ge–Te thin films prepared by combinatorial sputtering

**DOI:** 10.1038/s41598-021-91138-x

**Published:** 2021-06-03

**Authors:** C. Mihai, F. Sava, I. D. Simandan, A. C. Galca, I. Burducea, N. Becherescu, A. Velea

**Affiliations:** 1 National Institute of Materials Physics, 077125 Magurele, Romania; 2 Horia Hulubei National Institute of Physics and Nuclear Engineering, 077125 Magurele, Romania; 3 Apel Laser Ltd., Vanatorilor 25, 077135 Mogosoaia, Romania

**Keywords:** Glasses, Information storage

## Abstract

The lack of order in amorphous chalcogenides offers them novel properties but also adds increased challenges in the discovery and design of advanced functional materials. The amorphous compositions in the Si–Ge–Te system are of interest for many applications such as optical data storage, optical sensors and Ovonic threshold switches. But an extended exploration of this system is still missing. In this study, magnetron co-sputtering is used for the combinatorial synthesis of thin film libraries, outside the glass formation domain. Compositional, structural and optical properties are investigated and discussed in the framework of topological constraint theory. The materials in the library are classified as stressed-rigid amorphous networks. The bandgap is heavily influenced by the Te content while the near-IR refractive index dependence on Ge concentration shows a minimum, which could be exploited in applications. A transition from a disordered to a more ordered amorphous network at 60 at% Te, is observed. The thermal stability study shows that the formed crystalline phases are dictated by the concentration of Ge and Te. New amorphous compositions in the Si–Ge–Te system were found and their properties explored, thus enabling an informed and rapid material selection and design for applications.

## Introduction

Amorphous chalcogenides possess unique properties that enable a broad range of applications in phase change memories^1^(PCM), Ovonic threshold switches (selectors)^2^, solar cells^3^, or photonics^4^.

Amorphous materials structure lacks long-range translational order^5^. The term “amorphous” is generally used for non-crystalline solids that are obtained as thin films, flakes, nanoparticles by bottom-up approach (condensation of vapors on a cold substrate), or as powders by grinding the material in a ball mill. On the other hand, glasses are defined as those non-crystalline materials obtained by rapid solidification of their melt^6^, or, according to Phillips^7^ and Elliott^8^, as non-crystalline solids which have a glass transition.

Chalcogens are the chemical elements S, Se or Te, which belong to the group VI A (16) in the periodic table. Tellurium has a more pronounced metallic character and quite isotropic atomic bonds as opposed to the lighter chalcogens (selenium and sulfur). Therefore, tellurides are poor glass formers with an increased tendency towards crystallization or decomposition^9^. They display a reduced glass formation domain (GFD)^10^ or no GFD at all, leading to crystallization of the melts upon cooling^11^. Amorphous chalcogenides are in quasi-equilibrium or meta-stable^12^ states, which means that their properties cannot be uniquely determined thermodynamically by temperature and pressure, because they change with time. It is considered that after infinitely long storage, the amorphous material relaxes into the corresponding crystal. Therefore, material properties are dependent upon the preparation method, synthesis conditions and prehistory.

The disordered structure and covalent bonding enable the continuous compositional variation, leading to a huge number of possible compositions but also to different properties for a fixed composition. Hence, there is a vast combinatorial space which requires the use of high-throughput^13^ combinatorial preparation and measurement methods for appropriate exploration.

Combinatorial depositions have been employed lately for materials discovery and optimization in different fields such as sensors^14^, photovoltaics^15^, thermoelectrics^16^, inorganic^17^ and functional materials^18^. Several deposition techniques like magnetron co-sputtering^19–21^, chemical bath deposition^22^ or pulsed laser deposition^16^ are suitable to obtain material libraries. Magnetron co-sputtering is used for the combinatorial synthesis of materials libraries because it can cover a large compositional area in a single deposition^23^ and offers good atomic mixing. A key feature of this method is the possibility to obtain amorphous thin film compositions which are located beyond the GFD of bulk glasses^24^. An increased mobility in the surface layers is obtained when magnetron sputtering deposition takes place on substrates at a temperature around 85% of the glass transition temperature (T_g_). This allows for local atomic configurations that are not reachable by melt-quenching techniques and leads to the formation of very stable amorphous compositions^25^, which according to molecular dynamics simulations of melt quenching would require thousands of years of annealing^26,27^.

The Si–Ge–Te amorphous system has been little studied in literature. Feltz et al.^28^ found the GFD of this system near the tie line between GeTe_4_ and SiTe_4_. Some specific compositions such as Si_15_Ge_11_Te_74_ were identified as promising candidates for PCM^29^. Moreover, a possible link between the electrical properties and the flexible nature of the amorphous network was suggested^30^.

More recent studies^2,31^, are focused only on limited regions or tie-lines (i.e. Si_x_(GeTe_6_)_1-x_ or Ge_x_Si_x_Te_1-2x_) inside the GFD. In Si_x_(GeTe_6_)_1-x_^2^, the addition of Si to GeTe_6_ produces an increase in the crystallization temperature and a transition from Ovonic threshold switching to Ovonic memory switching with a higher threshold voltage. For Ge_x_Si_x_Te_1-2x_^31^, the existence of an intermediate phase between 7.5 at% < x < 9 at% is of interest for PCM, because for these compositions properties such as resistivity or optical contrast remain unchanged. No studies on extended regions outside GFD were performed so far.

In this study, we explore the compositional, structural and optical properties of the Si–Ge–Te amorphous system, beyond the GFD, by focusing on the variation of Te concentration on a large compositional interval. A combinatorial approach through magnetron co-sputtering for materials library synthesis is employed. To our knowledge this is the most extensive study in the Si–Ge–Te ternary chalcogenide system with respect to the covered range of compositions. Rutherford backscattering (RBS), grazing incidence X-ray diffraction (GIXRD) and spectroscopic ellipsometry have been used to map the properties of the resulting library. Also, annealing was employed for thermal stability analysis.

## Results and discussion

### Chemical composition of the combinatorial chalcogenide library

**Figure 1:**
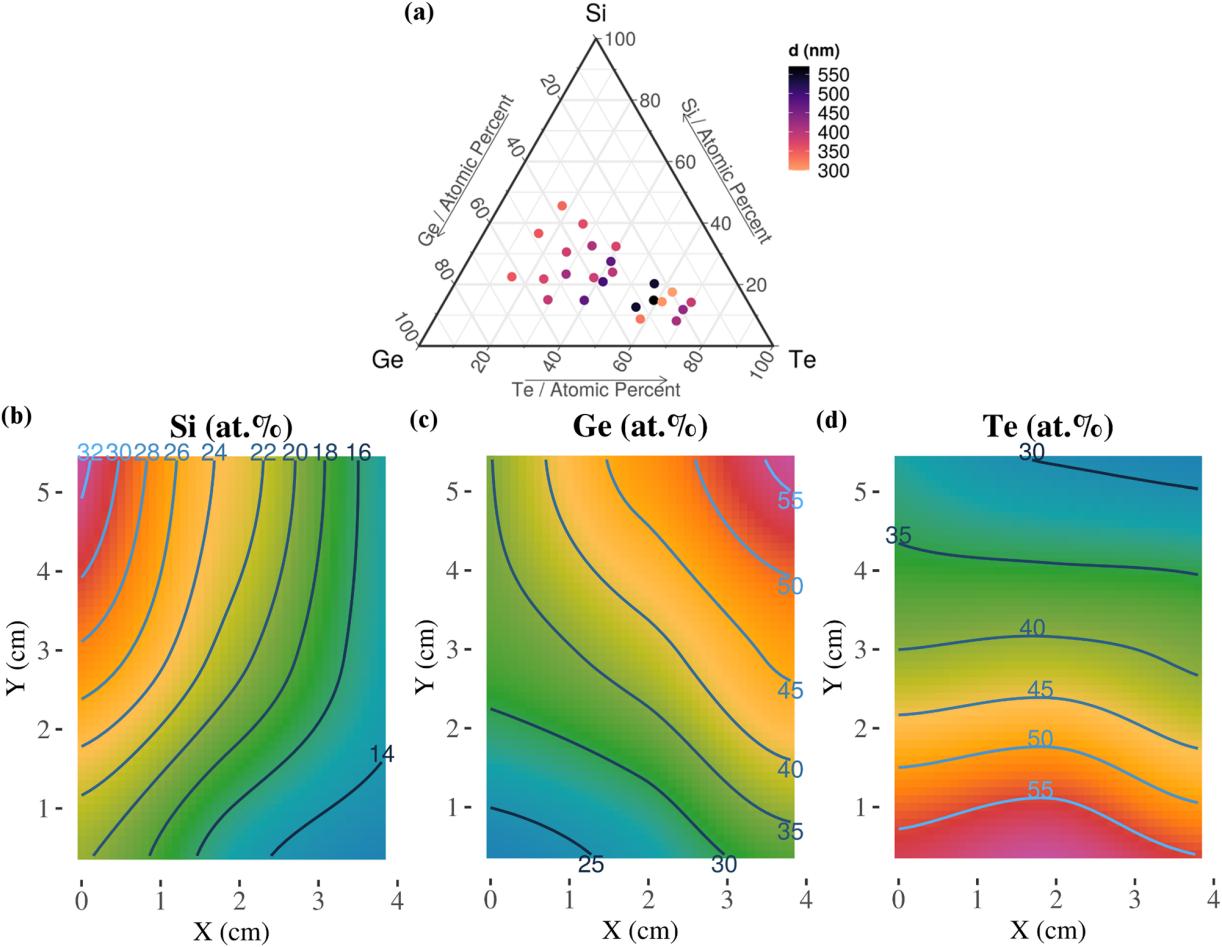
The chemical composition and thickness of the combinatorial chalcogenide library. (**a**) Thickness (*d*) distribution (measured by spectroscopic ellipsometry) in the center of the 24 samples; Elemental concentration gradient in one representative deposition for (**b**) Si; (**c**) Ge; (**d**) Te. The ternary diagram in (**a**) was generated with the R software [v. 3.6.3] (R: A language and environment for statistical computing, R Core Team, R Foundation for Statistical Computing, Vienna, Austria (2020) http://www.R-project.org/.) using the package ggtern [v. 3.3.0] (https://cran.r-project.org/web/packages/ggtern/index.html).

The combinatorial Si–Ge–Te library was deposited on 24 substrates using three sets of magnetron co-sputtering depositions. The library has the following compositional spread by element: [8.1 ÷ 45.6] at% for Si, [16 ÷ 62.5] at% for Ge and [15 ÷ 69.8] at% for Te. In comparison with other studies of the Si–Ge–Te system^28,30,31^, our new compositional domain is at least 5 times larger. An example of the concentration gradient from one deposition is given in Fig. 1b–d. The atomic composition is measured by RBS in the center of each sample with measurement uncertainty of less than 3 at%. The full composition map is obtained by spatial interpolation using a Locally Weighted Regression model^32^. Each point is estimated by fitting a low degree polynomial on a subset of the data, giving more weight to the points near the estimated point.

The thickness of the samples (Fig. 1a) is dependent on the deposition time and on the distance from the sputtering targets. The minimum thickness is 299.8 nm for Si_17.5_Ge_19.7_Te_62.8_ and the maximum is 570.5 nm for Si_14.9_Ge_26.3_Te_58.8_. The samples closest to the Te target are thicker due to the increased deposition rate for Te as compared with Si and Ge. The thickness of all the samples in the library, determined by spectroscopic ellipsometry, is given in Table 1. Both thickness and compositional lateral gradients are expected on each sample.

**Table 1:** Computed values of <*r*> *and* *n*_*c*_ from topological constraint theory and spectroscopic ellipsometry modelling parameters for the combinatorial library. The average coordination number, <*r*>, and the average number of constraints, *n*_*c*_, computed for the prepared compositions. Composition was determined using RBS measurements (< 3 at%). From spectroscopic ellipsometry analysis, the thickness (± 0.5 nm), *E*_*g*_ (± 0.01), *E*_0_ (± 0.02), *A* (± 1.0), *Γ* (± 0.1), ε_∞_ (± 0.02) and the values of the refractive indices at 405 nm (n_1_ ± 0.03), 587.6 nm (n_2_ ± 0.03) and 1550 nm (n_3_ ± 0.03) are tabulated (the given errors are the highest resulted from the fitting procedure).

Composition	<*r*>	*n*_*c*_	d (nm)	*E*_*g*_ (eV)	*E*_0_ (eV)	*A*	*Γ*	ε_∞_	n_1_	n_2_	n_3_
Si_22.5_Ge_62.5_Te_15_	3.70	6.25	352.9	0.80	4.1	87.7	5.5	9.6	3.3	3.6	3.2
Si_36.6_Ge_47.9_Te_15.5_	3.69	6.23	353.0	0.94	4.6	73.5	6.2	7.7	3.1	3.2	2.8
Si_45.6_Ge_36.8_Te_17.6_	3.65	6.12	339.5	1.02	4.5	61.5	5.6	7.6	3.0	3.0	2.7
Si_21.8_Ge_53.8_Te_24.4_	3.51	5.78	371.2	0.78	4.5	85.0	6.2	9.1	3.2	3.4	3.1
Si_30.6_Ge_43_Te_26.4_	3.47	5.68	380.8	0.87	4.8	76.5	6.8	7.8	3.0	3.2	2.9
Si_39.7_Ge_33.8_Te_26.5_	3.47	5.68	364.0	0.98	4.6	63.1	5.9	7.1	3.0	3.0	2.7
Si_15_Ge_56.1_Te_28.9_	3.42	5.55	391.3	0.71	3.7	101.1	5.1	11.8	3.4	3.9	3.6
Si_23.4_Ge_46.7_Te_29.9_	3.40	5.51	417.2	0.83	4.2	75.3	6.0	8.4	3.1	3.3	3.0
Si_32.6_Ge_34.8_Te_32.6_	3.35	5.37	407.8	0.88	4.6	67.5	6.3	7.3	2.9	3.1	2.8
Si_22.2_Ge_39.5_Te_38.3_	3.23	5.09	381.5	0.86	4.2	87.0	6.2	8.9	3.2	3.4	3.1
Si_14.8_Ge_45.9_Te_39.3_	3.21	5.04	471.5	0.79	4.1	92.2	5.9	10	3.3	3.6	3.3
Si_32.4_Ge_28.2_Te_39.4_	3.21	5.03	373.9	1.02	4.3	80.5	6.8	7.5	2.9	3.1	2.8
Si_27.5_Ge_32.1_Te_40.4_	3.19	4.98	472.1	0.98	3.8	76.1	6.1	7.5	2.8	3.1	2.8
Si_20.9_Ge_37.6_Te_41.5_	3.17	4.92	494.7	0.96	3.8	91.9	6.4	8.3	3.0	3.3	3.0
Si_24_Ge_33.3_Te_42.7_	3.15	4.87	397.3	0.98	4.1	87.9	6.7	8.1	3.0	3.3	2.9
Si_12.6_Ge_32.4_Te_55_	2.90	4.25	542.4	0.89	3.5	107.4	6.1	9.9	3.1	3.6	3.3
Si_20.3_Ge_23.4_Te_56.3_	2.87	4.18	539.1	0.95	3.6	98.0	6.0	8.9	3.0	3.4	3.1
Si_8.8_Ge_33.1_Te_58.1_	2.84	4.09	326.2	0.93	2.7	83.8	4.0	9.2	2.7	3.4	3.2
Si_14.9_Ge_26.3_Te_58.8_	2.82	4.06	570.5	0.92	3.3	102.0	5.6	9.5	3.0	3.5	3.2
Si_14.4_Ge_24.2_Te_61.4_	2.77	3.93	307.0	0.96	2.6	84.0	4.1	8.7	2.6	3.3	3.1
Si_17.5_Ge_19.7_Te_62.8_	2.74	3.86	299.8	0.95	2.5	72.5	3.4	8.3	2.4	3.2	3.0
Si_8.1_Ge_23.3_Te_68.6_	2.63	3.57	414.7	0.94	2.5	86.9	3.3	9.6	2.6	3.6	3.2
Si_11.8_Ge_19.5_Te_68.7_	2.63	3.57	431.5	0.91	2.4	86.3	3.1	9.8	2.5	3.5	3.3
Si_14.2_Ge_16_Te_69.8_	2.60	3.51	386.0	0.88	2.4	82.3	3.2	9.7	2.5	3.5	3.3

### Amorphous Si–Ge–Te formation domain

The glass formation domain (GFD) contains compositions that can be easily obtained in the bulk glassy state. The GFD of a system is usually mapped out systematically by sintering many samples of specific compositions which are reacted, quenched and then their crystalline or glassy phase is determined. The extent of GFD is dependent on the reacting temperature from which the melt is quenched, the quenching rate and the amount of material used in the sample preparation. For chalcogenide glasses it was shown that the glass-forming ability varies in the following order: S > Se > Te^33^. It was observed that near the border line of these domains one may find compositions with memory-switching properties^34^ (i.e. phase-change materials) or compositions with special thermal properties^35^ (i.e. intermediate phases).

**Figure 2:**
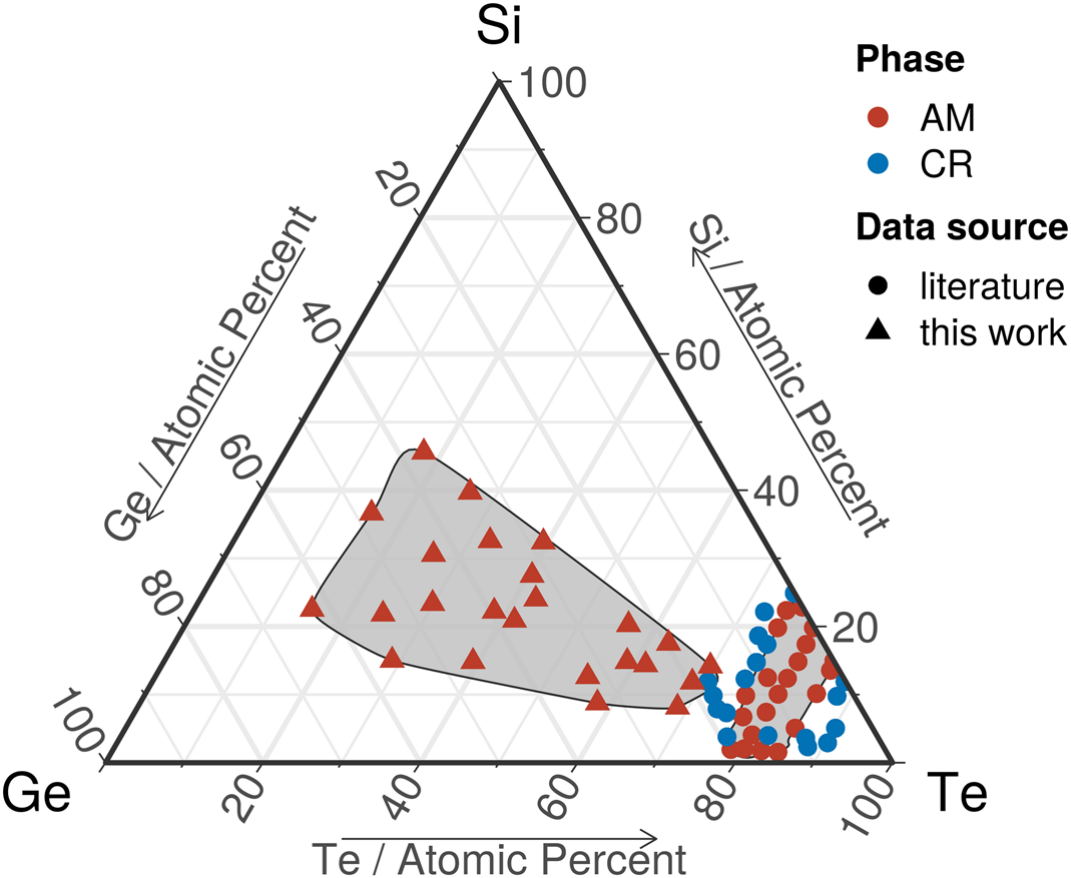
Si–Ge–Te ternary diagram. The structure of the as-deposited Si–Ge–Te library and the GFD from Ref.^28^. Amorphous (AM) compositions are shown in red color and crystalline (CR) samples are depicted in blue. Generated with the R software [v. 3.6.3] (R: A language and environment for statistical computing, R Core Team, R Foundation for Statistical Computing, Vienna, Austria (2020) http://www.R-project.org/.) using the package ggtern [v. 3.3.0] (https://cran.r-project.org/web/packages/ggtern/index.html).

In this study new amorphous Si–Ge–Te compositions are sought, so, the compositional space outside the GFD known from literature, is explored. The chemical composition of the 24 samples in the library, together with the GFD found by A. Feltz et al.^28^ are shown in the ternary diagram from Fig. 2.

The GIXRD measurements (data not shown) reveal that all the samples have amorphous structure.

### Topological constraint theory

Topological constraint theory^36,37^ classifies amorphous networks into flexible and rigid. The strong covalent bonds determine the local atomic structure of the disordered materials. In covalent solids, each atom supports two types of bonding constraints: bond-stretching constraints and bond-bending constraints. In an amorphous covalent network, the average number of bond-stretching (*n*_*BS*_) and the average number of bond-bending (*n*_*BB*_) constraints per atom (averaged over all chemical elements of the material) can be computed using the average coordination number (<*r*>) over all atoms in the amorphous covalent network: *n*_*BS*_ =  <*r*>*/*2 and *n*_*BB*_ = 2 <*r*>–3. If the average number of constraints per atom, *n*_*c*_ (*n*_*c*_ = *n*_*BS*_ + *n*_*BB*_), in an amorphous covalent material, is less than 3 (<*r*> < 2.4), its network is considered flexible, the bonds between atoms are very flexible and the material is unstable because of entropy. If *n*_*c*_ is greater than 3 (<*r*> > 2.4), the network is stressed-rigid**,** the atomic bonds are very rigid, and the amorphous covalent material is unstable because of strain. When *n*_*c*_ = 3 (<*r*> = 2.4), which is equal to the number of degrees of freedom per atom in three dimensions, the network is called isostatic, and the amorphous covalent material is stable.

Amorphous chalcogenides are mostly covalently bonded, which allows for the continuous variation of their composition in atomic ratios, rather than in chemical units as in the case of oxides, which have more ionic bonds. The average coordination number <*r*> per atom, is a simple yet powerful topological descriptor for understanding the compositional variation in amorphous chalcogenides. We notice however, that this descriptor does not take into account the chemical nature of bonds or the medium-range order. As an example, for the Si_x_Ge_y_Te_1-x–y_ composition, <*r*> is computed as [4*x* + 4*y* + 2(1* − x − y*)], considering that Ge and Si are fourfold coordinated and Te is twofold coordinated, according to the “8*-N* rule” (*N* being the number of outer shell electrons). However, for Te-based compositions, the average coordination number might not be an accurate descriptor due to the metallic character of Tellurium, whose coordination number (n_Te_) can deviate from the 8*-N* rule (n_Te_ > 2). For example, in the ternary Si_10_Ge_10_Te_80_ glass, it was found that n_Si_ = 3.87, n_Ge_ = 3.91 and n_Te_ = 2.39^31^. Nevertheless, topological constraint theory is a powerful tool to understand Te-based amorphous chalcogenides.

**Figure 3:**
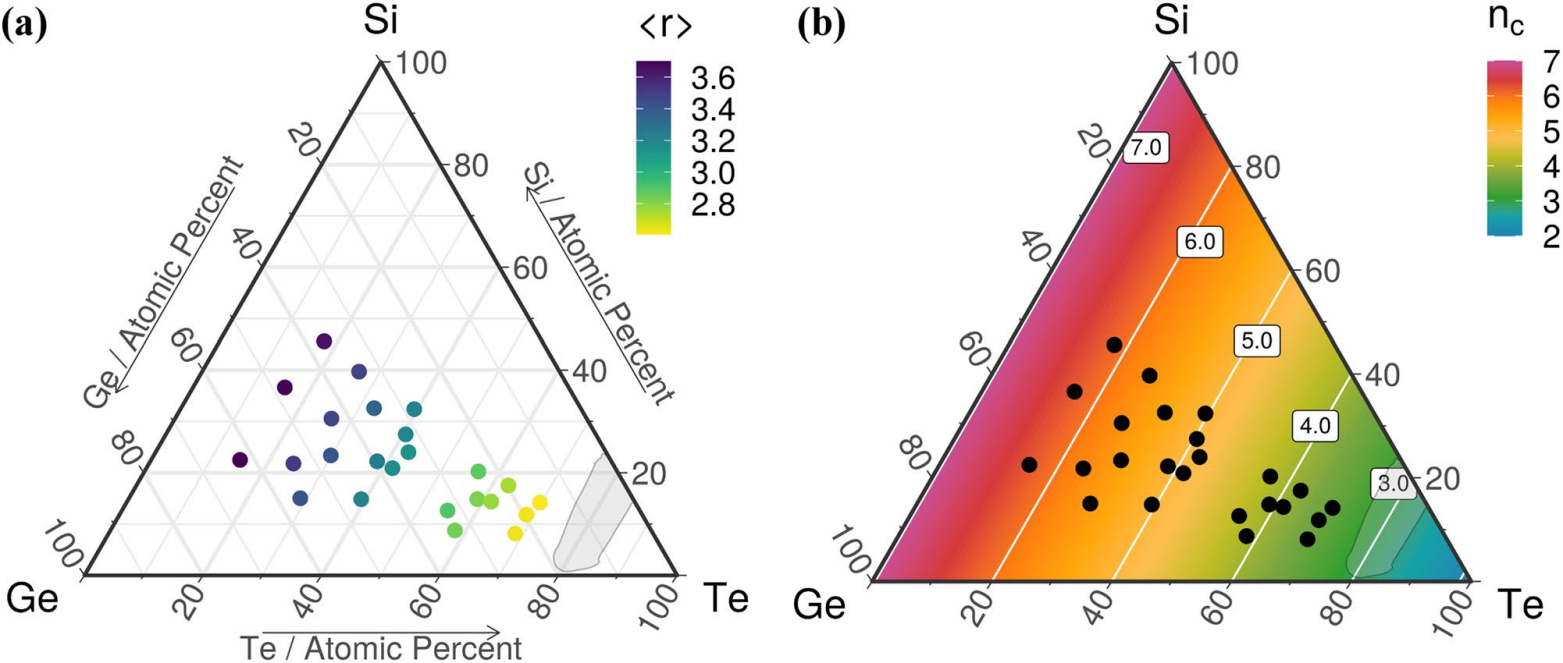
Topological constraint theory parameters for the Si–Ge–Te system. (**a**) The average coordination number and (**b**) the average number of constraints. In gray is depicted the glass formation domain. Ternary diagrams were generated with the R software [v. 3.6.3] (R: A language and environment for statistical computing, R Core Team, R Foundation for Statistical Computing, Vienna, Austria (2020) http://www.R-project.org/.) using the packages ggtern [v. 3.3.0] (https://cran.r-project.org/web/packages/ggtern/index.html) and directlabels [v. 2020.6.17] (https://cran.r-project.org/web/packages/directlabels/index.html).

In the Si–Ge–Te system (Fig. 3a), the maximum value of <*r*> is 3.7 for Si_22.5_Ge_62.5_Te_15_, which is the least stable material in the library. As we approach the GFD, the concentration of Te increases and the network becomes more flexible. The average coordination number decreases toward the optimal value of 2.4 for stable glasses. The sample closest to this magic number, namely Si_14.2_Ge_16_Te_69.8_, has an <*r*> of 2.6. The <*r*> values for the entire library are given in Table 1. A map of the flexible and stressed-rigid regions is shown in Fig. 3b. The average number of constraints increases as we move away from the Te vertex (where *n*_*c*_ = 2), towards the tie line between Si and Ge (where *n*_*c*_ = 7). The compositional join line *n*_*c*_ = 3 (tie line between GeTe_4_ and SiTe_4_) crosses through the middle of the GFD. An important composition for OTS, GeTe_6_, is near this compositional join line, whereas the stiffness threshold in the Ge_x_Te_100–x_ system is found at *x* = 20 at%. Also, phase change materials are usually found in the stressed-rigid area of the map, for instance GeTe has *n*_*c*_ = 4.5. All the samples in the library are located in the stressed-rigid region (*n*_*c*_ > 3), with *n*_*c*_ in the interval 3.5 to 6.2.

### Optical properties

Due to their applications in optical data storage^38^ and optical sensors^39^, the optical properties of amorphous chalcogenide materials are of great interest for the digital era. Spectroscopic ellipsometry is a sensitive technique used to measure  the thickness and optical constants of amorphous and crystalline chalcogenide materials^40,41^.

**Figure 4:**
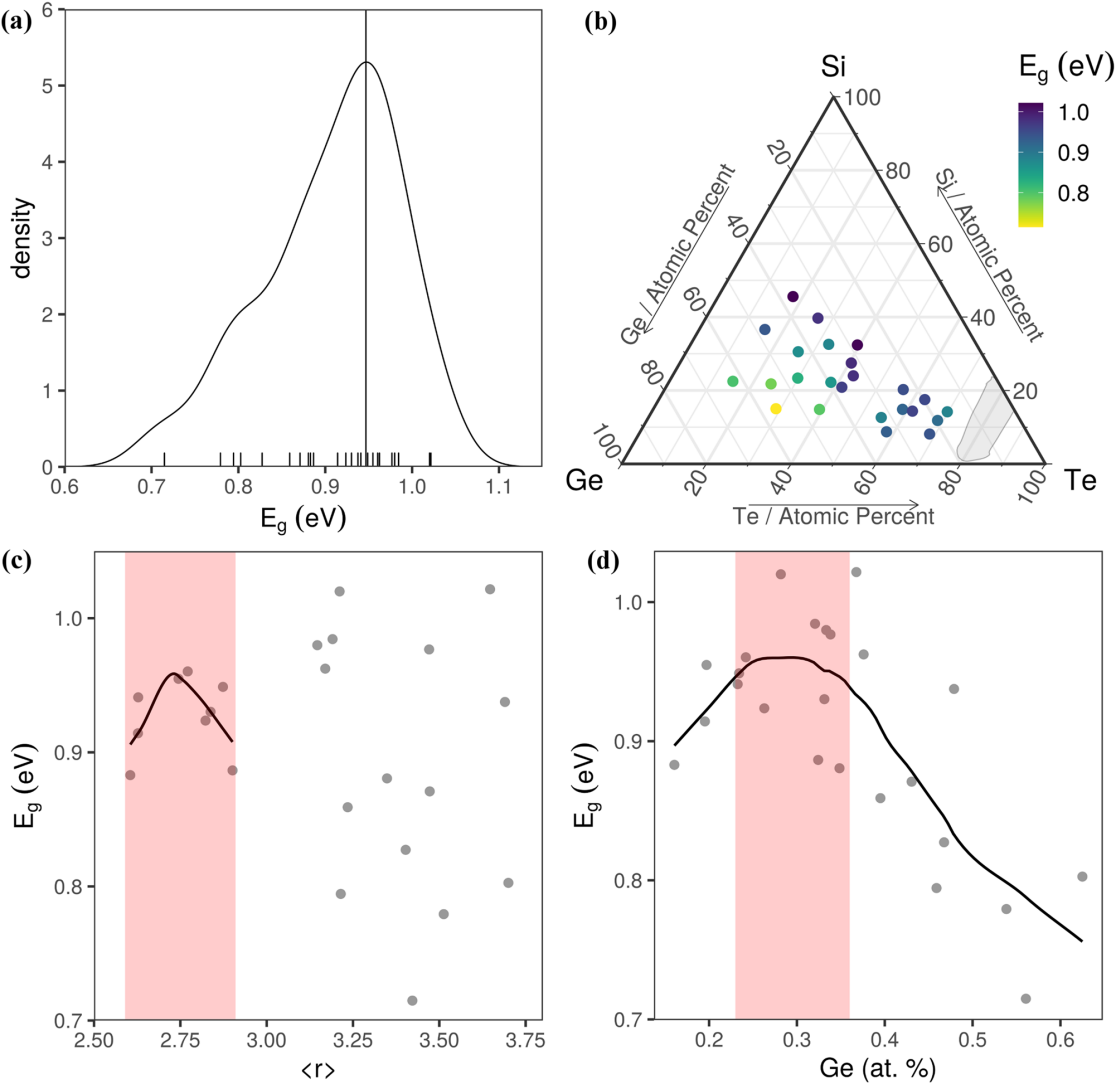
Variation of bandgap in the Si–Ge–Te system. (**a**) The probability density function of *E*_*g*_; Bandgap as a function of: (**b**) composition, (**c**) the average coordination number, (**d**) Ge concentration. The black lines and red shaded areas in (**c**) and (**d**) are guides to the eye. The ternary diagram in (**b**) was generated with the R software [v. 3.6.3] (R: A language and environment for statistical computing, R Core Team, R Foundation for Statistical Computing, Vienna, Austria (2020) http://www.R-project.org/.) using the package ggtern [v. 3.3.0] (https://cran.r-project.org/web/packages/ggtern/index.html).

**Figure 5:**
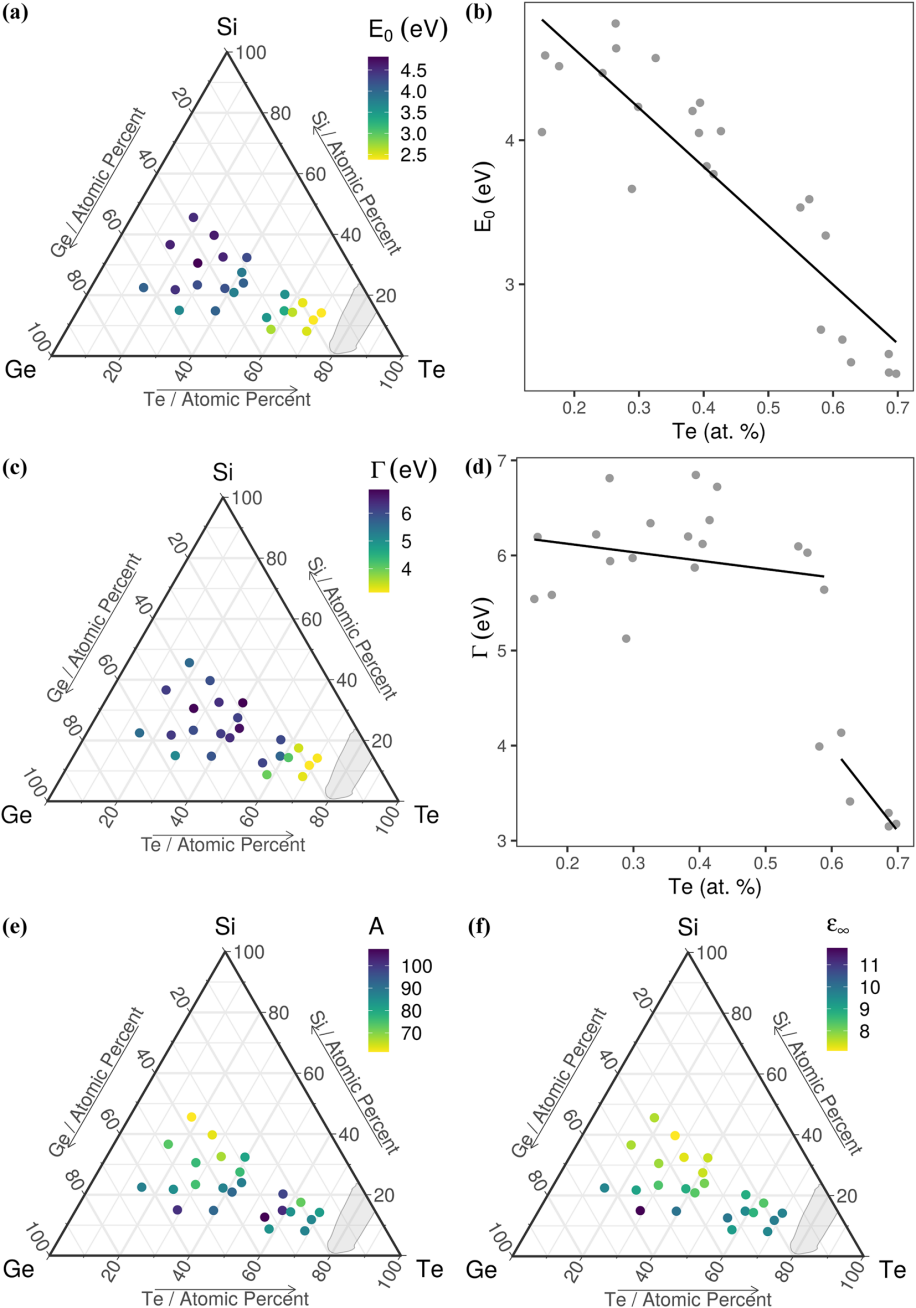
Spectroscopic ellipsometry parameters for the Si–Ge–Te library. Resonance energy, *E*_0_, as a function of (**a**) composition and (**b**) Te concentration; Broadening parameter of the oscillator, *Γ*, as a function of (**c**) composition and (**d**) Te concentration; (**e**) Amplitude, A, as a function of composition; and (**f**) Optical dielectric constant, ε_∞_, as a function of composition. The black line in (**b**) and (**d**) is a guide to the eye. Ternary diagrams were generated with the R software [v. 3.6.3] (R: A language and environment for statistical computing, R Core Team, R Foundation for Statistical Computing, Vienna, Austria (2020) http://www.R-project.org/.) using the package ggtern [v. 3.3.0] (https://cran.r-project.org/web/packages/ggtern/index.html).

The extracted values of the parameters used to model the experimental data are shown in Figs. 4, 5 and in Table 1. All the fitted ellipsometry data are shown in Supplementary Figure S2. The mean squared errors (MSE) values we have obtained in the present study are between 6 and 68. It seems that MSE increases as the Si quantity increases.

*E*_*g*_ in Si–Ge–Te is determined by the concentration of both cation and anion species. Most of the amorphous telluride materials in the library have an *E*_*g*_ around 0.95 eV, as can be seen in Fig. 4a. Jovari et al*.*^11^ showed that in thermally co-evaporated amorphous Ge_x_Te_100-x_ alloys (12 ≤ x ≤ 44.6) the majority of atoms obey the 8*-N* rule, but there are homopolar bonds which evolve monotonically with increasing x (the percent of Te–Te bonds decreases from 73.4 to 35.9%, while the percent of Ge–Ge bonds increases from 1 to 56.6%), resulting in chemical disorder. This suggests that the Te, Si and Ge nano-clusters can make an important contribution to the optical band gap of amorphous Si–Ge–Te compositions.

According to K. Tanaka^12^, the width of the conduction band is proportional with the average coordination number <*r*> , while the width of the valence band is inversely proportional to the length of van der Waals bonds in chalcogenide glasses. Considering that the difference between the centers of the conduction and valence bands is constant (simple tight binding electrons model), the dependence of *E*_*g*_ on <*r*> shows a minimum at <*r*> = 2.4 due to an increase of the conduction band width which produces a decrease in *E*_*g*_. Then *E*_*g*_ increases, when <*r*> is between 2.4 and 2.67, due to the increase in the van der Waals bonds which produces a decrease in the width of the valence band and therefore the increase in *E*_*g*_. A similar behaviour was observed in covalent chalcogenide glasses^42^. The small shifts from these values, observed in the Si–Ge–Te system, Fig. 4c, are produced by the ionic and metallic character of telluride materials. The variation of the bandgap with the average coordination number in Fig. 4c, shows traces of a minimum at small <*r*>. A peak in the bandgap is observed for <*r*> ~ 2.75, which echoes the maximum at 2.67 observed in other chalcogenide glasses^43,44^. The value of 2.67 is related to planar network glasses, where the angular constraints (*n*_*BB*_) are reduced from 2 <*r*>–3 to <*r*>–1, resulting in an average coordination number of 2.67 when *n*_*c*_ = 3. After this peak, the bandgap decreases again due to the increase in the width of the conduction band. Above 3, there is no clear dependence of *E*_*g*_ on <*r*>.

Figure 4d shows the variation of the bandgap with the Ge concentration. We can observe that the bandgap has a maximum between 23 and 36 at% Ge. A similar dependence was observed by C. Vigreux et al.^45^ in Ge-Te films. These singularities in bandgap can also be linked to nanophase separation as suggested by Boolchand et al*.*^46^.

The critical energy, *E*_0_, shown in Fig. 5a, has a minimum value of 2.4 eV for Si_11.8_Ge_19.5_Te_68.7_ and Si_14.2_Ge_16_Te_69.8_, and a maximum of 4.8 eV for Si_30.6_Ge_43_Te_26.4_. For energies above the bandgap, electronic critical point transitions in the dielectric function are present due to electron transitions between the valence and conduction bands^47^. In chalcogenides, the upper edge of the valence band is composed of lone pair states^48^, so above the bandgap there are transitions from these states to the conduction band. *E*_0_ is linearly dependent on the Te concentration. As the Te concentration increases, the resonance energy decreases (Fig. 5b).

The broadening parameter is scaling inversely with the lifetime of carriers excited into deep states in the conduction and valence bands^49^. If the broadening is small, it indicates a more ordered material with less scattering. Strain and defects in crystalline networks, lead to a higher broadening and lower mean free path of carriers. In amorphous networks there are similar defects in the continuous random network. In the Si–Ge–Te system, as the compositions approach the GFD, the average coordination number decreases and the structural order increases (bond lengths and bond angles distributions are narrower), which is observed by the decrease of *Γ* (Fig. 5c). The most ordered composition is Si_11.8_Ge_19.5_Te_68.7_ with *Γ* = 3.1, while the least ordered are Si_30.6_Ge_43_Te_26.4_ and Si_32.4_Ge _28.2_Te_39.4_ with *Γ* = 6.8. An abrupt transition, from disorder (*Γ* ~ 6) to order (*Γ* ~ 4), is observed when the Te concentration reaches 60 at%, while above this value the decrease is linear (Fig. 5d). The values obtained for *Γ* are comparable to those of other amorphous chalcogenide materials^40,41,50^. The abrupt drop in the broadening parameter usually accompanies structural changes and was observed for Te-based chalcogenides such as Ge–Te^41^ and Ge–Sb–Te^51^. Moreover, a systematic decrease of *Γ* with increasing chalcogenide content in glasses was also observed by Orava et al^52^. The amplitude, A, Fig. 5e, has the minimum value of 61.5 for Si_45.6_Ge_36.8_Te_17.6_, the sample with the highest concentration of Si, and the maximum value of 107.4 for Si_12.6_Ge_32.4_Te_55_.

ε_∞_, which is linked to the degree of polarization, is shown in Fig. 5f. Below the bandgap, the dielectric function is governed by the electronic polarizability of the valence electrons^53^. ε_∞_ for Te is 11 for the amorphous phase^54^, which is in accordance with the values obtained by us. It increases with the increase of Te concentration and the decrease of Si.

**Figure 6:**
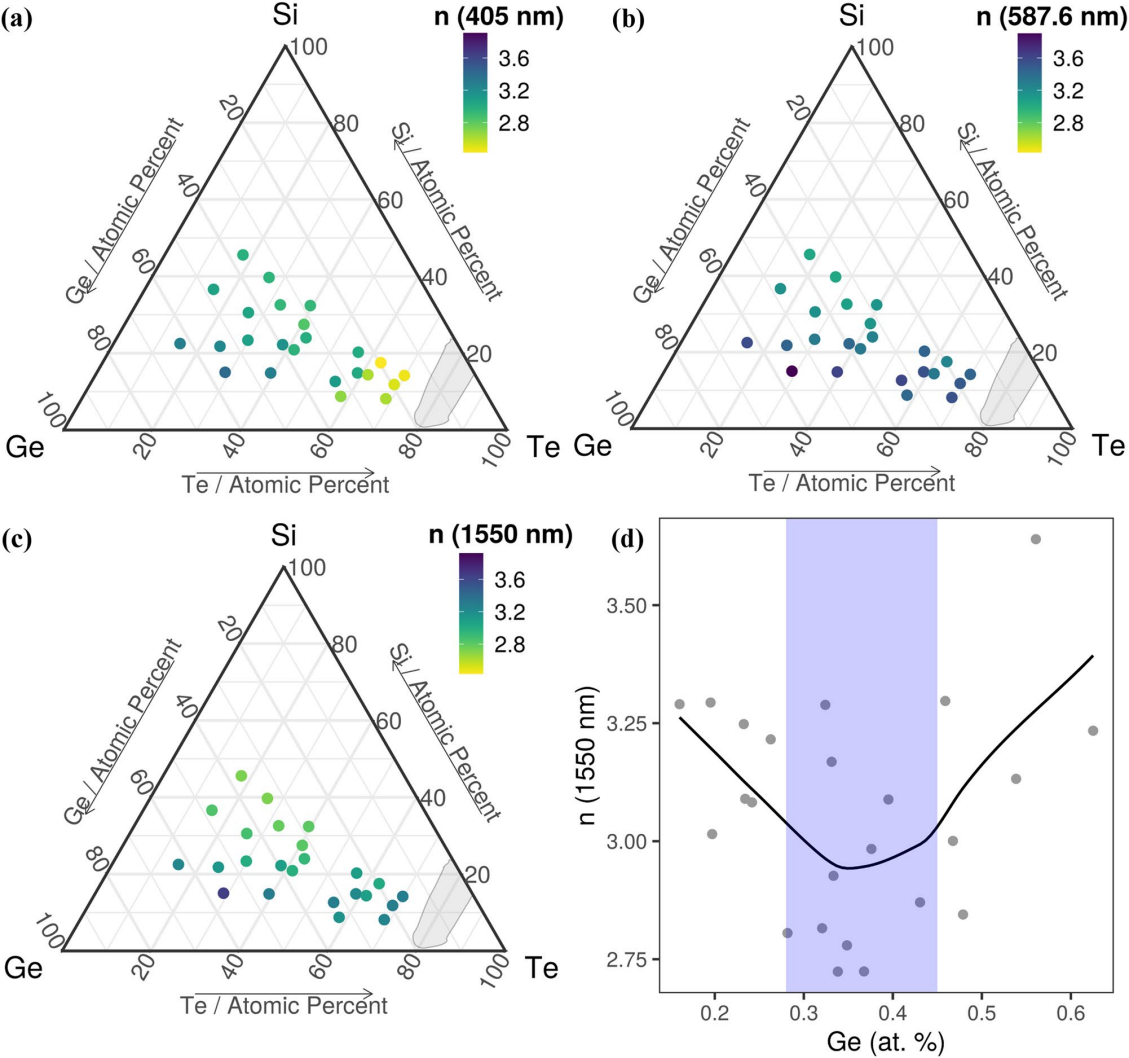
The refractive index at different wavelengths: (**a**) 405 nm; (**b**) 587.6 nm; (**c**) 1550 nm and (**d**) The variation of the near-IR refractive index as a function of Ge concentration. The black line and blue shaded area in (**d**) are guides to the eye. Ternary diagrams were generated with the R software [v. 3.6.3] (R: A language and environment for statistical computing, R Core Team, R Foundation for Statistical Computing, Vienna, Austria (2020) http://www.R-project.org/.) using the package ggtern [v. 3.3.0] (https://cran.r-project.org/web/packages/ggtern/index.html).

In the visible domain, chalcogenide glasses are generally characterized by the value of their refractive index, *n*, near the center of the domain (587.6 nm at the *d* spectral line of He). Chalcogenide amorphous thin films are important in the production of near-IR (1550 nm) integrated optical devices for the detection of biological or environmental variations^39^ and Blu-ray discs (BD)^1^. The refractive indices at 405 nm (the wavelength used for BD), at 587.6 nm (visible region) and 1550 nm (near-IR region) are shown in Fig. 6 and Table 1. The extinction coefficient for the same wavelengths is shown in Fig. S3. The Te percent has a large influence on the refractive index, which increases when the Te concentration increases. For low wavelengths (405 nm, Fig. 6a), the refractive index has a minimum close to the GFD (the composition with the lowest refractive index is Si_17.5_Ge_19.7_Te_62.8_ with n = 2.4**)**, whereas for higher wavelengths (587.6 nm and 1550 nm, Fig. 6b,c) the trend reverses and the refractive index increases near GFD.

The refractive index at 1550 nm has a minimum in the region between 28 and 45 at% of Ge (Fig. 6d). Such a minimum is very important in near-IR waveguides applications, because it shows that the refractive index can be controlled by the Ge concentration. The optical properties of the combinatorial Si–Ge–Te library, such as the bandgap and the refractive index, can be gradually modified with composition and show important extrema in their dependence.

### Thermal stability

The glass transition temperature, *T*_*g*_, is defined as the transition temperature from a glassy state to a supercooled liquid, viscous state^12^. At high temperatures (higher than the melting temperature), the liquid has the configuration with the highest enthalpy, whereas at room temperature, the crystal has the smallest enthalpy. Instead, at room temperature, the glass has a local minimum enthalpy, that corresponds to a quasi-equilibrium or metastable state. So, the glass transition is a kinetic phenomenon where structural relaxation occurs. Structural relaxation consists of atomic rearrangements that allow the material to reach the equilibrium state at a given temperature.

**Figure 7:**
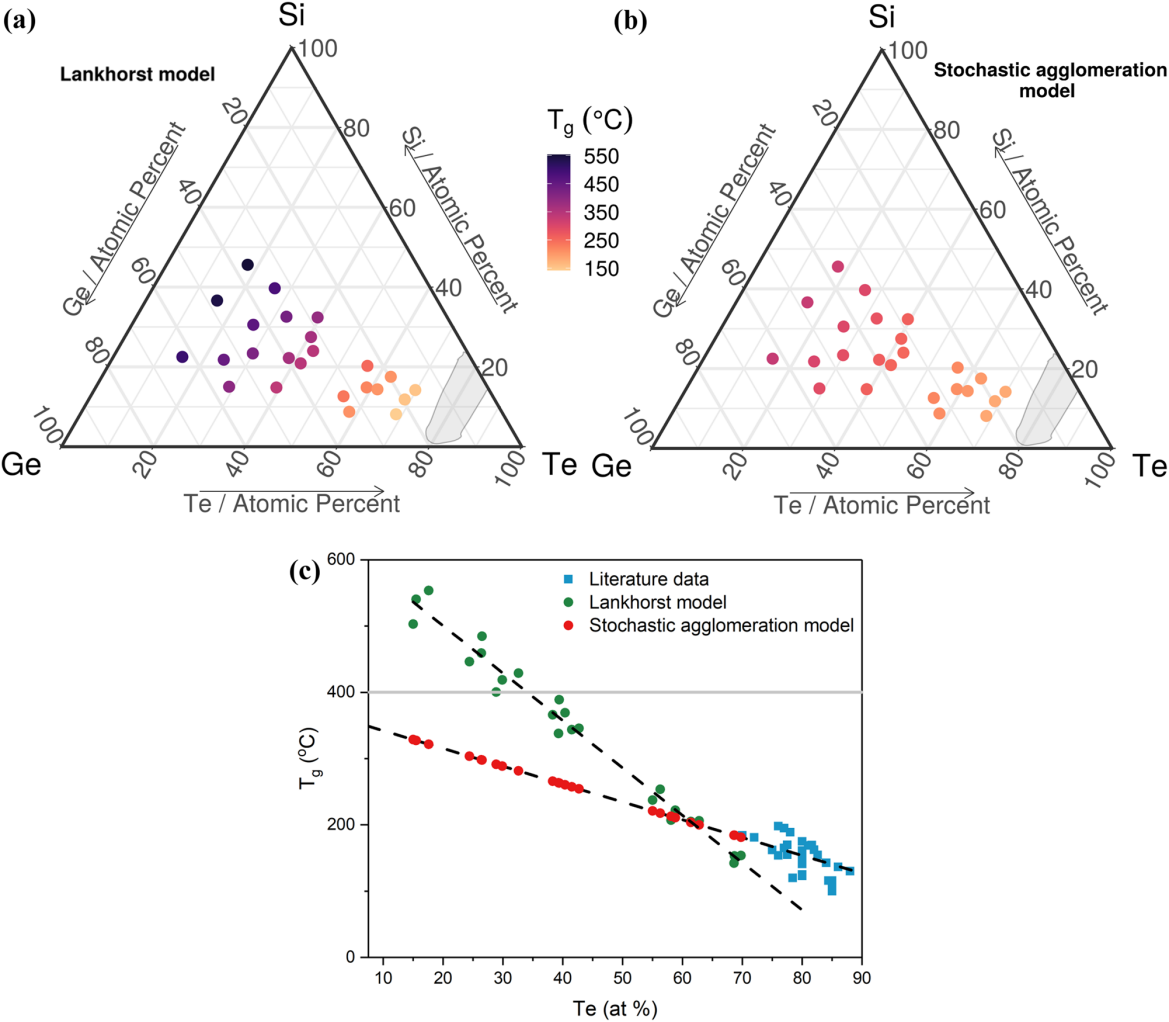
Glass transition temperature of the Si–Ge–Te library. Ternary diagrams showing (**a**) *T*_*g*_ computed with the Lankhorst model^56^ and (**b**) the stochastic agglomeration model^57,58^, for the sputtered library. (**c**) *T*_*g*_ as a function of Te concentration, for arbitrary Si and Ge concentration. Computed values for Lankhorst model (**a**) are shown as green dots, and as red dots for the Stochastic agglomeration model (**b**). The blue squares show experimental data from literature^28,31^. The black dashed lines are guides to the eye. Ternary diagrams were generated with the R software [v. 3.6.3] (R: A language and environment for statistical computing, R Core Team, R Foundation for Statistical Computing, Vienna, Austria (2020) http://www.R-project.org/.) using the package ggtern [v. 3.3.0] (https://cran.r-project.org/web/packages/ggtern/index.html).

Te-based materials should crystallize above the glass transition temperature^55^, therefore the glass transition temperature can be considered the lower limit for crystallization^2^. Two models are used to estimate *T*_*g*_. First, using the Lankhorst model, the computed *T*_*g*_ values, are in the interval 142.1 °C (for Si_8.1_Ge_23.3_Te_68.6_) ÷ 553.5 °C (for Si_45.6_Ge_36.8_Te_17.6_) for compositions with the lowest and highest amount of Si, respectively, and are shown in Fig. 7a, and Table 2. Second, the obtained *T*_*g*_ values, from the Stochastic agglomeration model, are between 181.4 °C (for Si_14.2_Ge_16_Te_69.8_) ÷ 328.7 °C (for Si_22.5_Ge_62.5_Te_15_) for the compositions with the highest and lowest amount of Te, respectively, and are presented in Fig. 7b and Table 2.

**Table 2:** Crystalline phases in the annealed Si–Ge–Te library and computed *T*_*g*_ values. The crystalline phases formed after annealing at 400 °C and the glass transition temperatures, computed using the Lankhorst, *T*_*g*_^*L*^, and the Stochastic agglomeration model, *T*_*g*_^*S*^, for the prepared Si–Ge–Te library. The indicated compositions are measured in the center of each sample. Notation: s.g. = space group.

Composition	Crystalline phase	*T*_*g*_^*L*^ (°C)	*T*_*g*_^*S*^ (°C)
Si_22.5_Ge_62.5_Te_15_	Rhombohedral Ge_0.99_Te_1.01_, PDF 04-002-6696, s.g. R3m (160)	503.0	328.7
Si_36.6_Ge_47.9_Te_15.5_	Rhombohedral Ge_0.99_Te_1.01_, PDF 04-002-6696, s.g. R3m (160)	540.1	327.4
Si_45.6_Ge_36.8_Te_17.6_	Rhombohedral Ge_0.99_Te_1.01_, PDF 04-002-6696, s.g. R3m (160)	553.5	321.6
Si_21.8_Ge_53.8_Te_24.4_	Rhombohedral Ge_0.99_Te_1.01_, PDF 04-002-6696, s.g. R3m (160)	446.1	303.5
Si_30.6_Ge_43_Te_26.4_	Hexagonal Te, PDF 03-065-2270, s.g. P3121 (152) Rhombohedral Ge_0.978_Te, PDF 04-002-5662, s.g. R3m (160)	459.2	298.1
Si_39.7_Ge_33.8_Te_26.5_	Hexagonal Te, PDF 03-065-2270, s.g. P3121 (152) Rhombohedral Ge0.976Te, PDF 04-002-5568, s.g. R3m (160)	484.8	297.8
Si_15_Ge_56.1_Te_28.9_	Rhombohedral GeTe, PDF 00-047-1079, s.g. R3m (166) Hexagonal Te, PDF 00-036-1452, s.g. P3121 (152)	400.5	291.3
Si_23.4_Ge_46.7_Te_29.9_	Rhombohedral GeTe, PDF 00-047-1079, s.g. R3m (166)	418.6	288.7
Si_32.6_Ge _34.8_Te_32.6_	Rhombohedral GeTe, PDF 00-047-1079, s.g. R3m (166)	429.0	281.4
Si_22.2_Ge_39.5_Te_38.3_	Hexagonal Te, PDF 03-065-2270, s.g. P3121 (152)	366.2	266.1
Si_14.8_Ge_45.9_Te_39.3_	Rhombohedral GeTe, PDF 00-047-1079, s.g. R3m (166)	338.0	263.4
Si_32.4_Ge_28.2_Te_39.4_	Hexagonal Te, PDF 03-065-2270, s.g. P3121 (152)	388.7	263.0
Si_27.5_Ge_32.1_Te_40.4_	Hexagonal Te, PDF 00-036-1452, s.g. P3121 (152)	369.0	260.2
Si_20.9_Ge_37.6_Te_41.5_	Hexagonal Te, PDF 00-036-1452, s.g. P3121 (152) Rhombohedral GeTe, PDF 00-047-1079, s.g. R3m (166)	343.8	257.4
Si_24_Ge_33.3_Te_42.7_	Hexagonal Te, PDF 03-065-2270, s.g. P3121 (152)	345.6	254.3
Si_12.6_Ge_32.4_Te_55_	Hexagonal Te, PDF 00-036-1452, s.g. P3121 (152) Rhombohedral GeTe, PDF 00-047-1079, s.g. R3m (166)	237.5	221.2
Si_20.3_Ge_23.4_Te_56.3_	Hexagonal Te, PDF 00-036-1452, s.g. P3121 (152)	253.7	217.5
Si_8.8_Ge_33.1_Te_58.1_	Rhombohedral GeTe, PDF 00-047-1079, s.g. R3m (166) Hexagonal Te, PDF 00-036-1452, s.g. P3121 (152)	207.2	212.7
Si_14.9_Ge_26.3_Te_58.8_	Hexagonal Te, PDF 00-036-1452, s.g. P3121 (152) Rhombohedral GeTe, PDF 00-047-1079, s.g. R3m (166)	222.0	210.7
Si_14.4_Ge_24.2_Te_61.4_	Hexagonal Te, PDF 04-007-5290, s.g. P3121 (152)	204.9	203.8
Si_17.5_Ge_19.7_Te_62.8_	Hexagonal Te, PDF 04-007-5290, s.g. P3121 (152)	206.2	200.2
Si_8.1_Ge_23.3_Te_68.6_	Hexagonal Te, PDF 04-007-5290, s.g. P3121 (152)	142.1	184.5
Si_11.8_Ge_19.5_Te_68.7_	Hexagonal Te, PDF 03-065-3370, s.g. P3121 (152)	153.0	184.4
Si_14.2_Ge_16_Te_69.8_	Hexagonal Te, PDF 04-007-5290, s.g. P3121 (152)	153.7	181.4

In Fig. 7c, the predictions of the two models, namely the Lankhorst^56^ model and the stochastic agglomeration model^57,58^, used to estimate *T*_*g*_, are compared. As a general observation, both models predict that *T*_*g*_ decreases with the increase of Te concentration. It is known that *T*_*g*_ rises with the increase of silicon content in Si–Te glasses, and by substituting germanium for silicon, the *T*_*g*_ values first increase and then decrease^28^. The Lankhorst model underestimates^55^ *T*_*g*_, as we also observe in Fig. 7c for high Te concentrations. Both models predict a similar transition temperature for Si_14.4_Ge_24.2_Te_61.4._ (*T*_*g*_^*L*^ = 204.9 °C and *T*_*g*_^*S*^ = 203.8 °C). Also, for the three compositions on the tie line between Ge_2_Te_3_ and Si_2_Te_3_ (Si_8.8_Ge_33.1_Te_58.1_, Si_14.9_Ge_26.3_Te_58.8_ and Si_17.5_Ge _19.7_Te_62.8_) the predictions of the two models are very close (difference < 15 °C). At low Te concentrations (below 35 at%), Lankhorst’s model predicts that 9 of the samples should have a *T*_*g*_ higher than 400 °C (the first nine compositions in Table 2).

**Figure 8:**
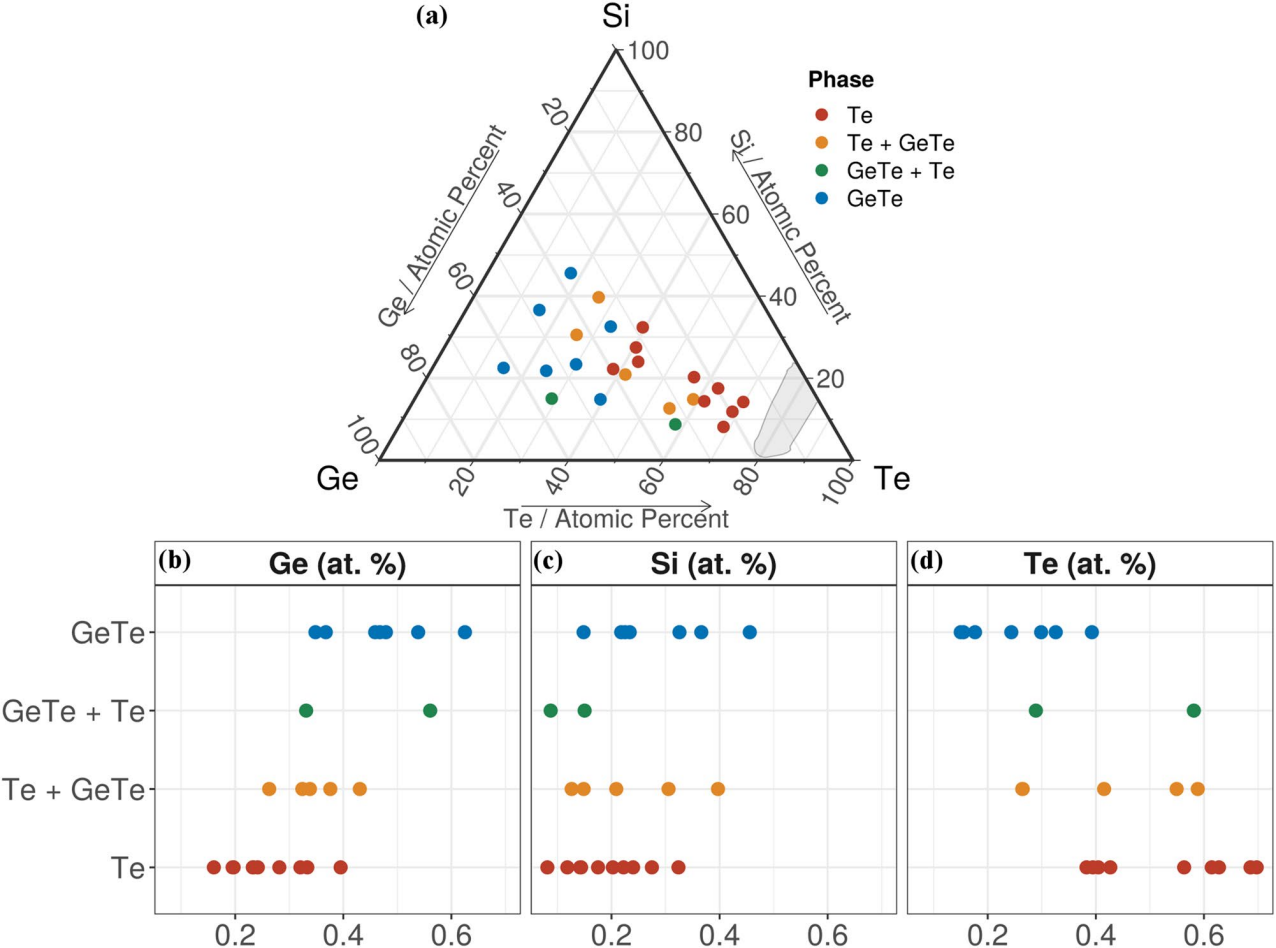
Crystalline phases formed after annealing the combinatorial library at 400 °C. Crystalline phases shown as a function of (**a**) composition in the Si–Ge–Te ternary diagram; and elemental concentration of: (**b**) Ge; (**c**) Si and (**d**) Te. The ternary diagram in (**a**) was generated with the R software [v. 3.6.3] (R: A language and environment for statistical computing, R Core Team, R Foundation for Statistical Computing, Vienna, Austria (2020) http://www.R-project.org/.) using the package ggtern [v. 3.3.0] (https://cran.r-project.org/web/packages/ggtern/index.html).

In order to test the thermal stability of the amorphous films and to see which model gives more accurate *T*_*g*_ estimates, the samples were annealed at 400 °C. Moreover, for some applications, such as OTS^2^, the amorphous phase should meet the processing needs of back-end-of-line (BEOL) integration, so this test should also show which compositions in the library satisfy this requirement. Since the glass transition temperature can be considered the lower limit for crystallization^2^, if the compositions stay amorphous up to 400 °C, then the Lankhorst model predicts well *T*_*g*_, otherwise if the samples crystallize below this temperature, then the stochastic agglomeration model is more precise. The results are shown in Fig. 8a, where it can be observed that all samples are crystalline at 400 °C. In conclusion, the stochastic agglomeration model is more accurate in predicting the glass transition temperatures.

Based on the crystalline phases which are formed, several groups can be distinguished in the Si–Ge–Te ternary diagram (Fig. 8a). The GIXRD patterns of all our samples before and after annealing are shown in Supplementary Fig. S1.

The first group (blue dots in Fig. 8a) contains the samples with the lowest concentration of Te (less than 40 at%, see also Fig. 8d). These samples crystallize in the rhombohedral germanium telluride phases (space group R3m (160) or R-3 m (166), PDF # 04–002-6696, PDF # 04–002-5568, PDF # 04–002-5568 or PDF # 00–047-1079). These are GeTe phases or GeTe phases with a small Te excess (see Table 2). The remaining Ge and Si form, most probable, a minority Ge_x_Si_1-x_ amorphous phase.

The second group (green dots in Fig. 8a) contains two samples which crystallize in majority in rhombohedral GeTe (space group R3m (160) or R-3 m (166), PDF 01–076-7106 and PDF # 00–047-1079) and in minority in hexagonal Te (space group P3121 (152), PDF # 00–036-1452). These are samples with a lower concentration of Si (less than 15 at%, see also Fig. 8c) than the first group. Small quantities of amorphous Ge_x_Si_1-x_ are also present in these samples.

The third group (orange dots in Fig. 8a) is composed of hexagonal Te (space group P3121 (152), PDF 00–036-1452) in majority and rhombohedral GeTe in minority (space group R3m (160) or R-3 m (166), PDF # 04–002-5568, PDF # 04–002-5662 or PDF # 00–047-1079).

The last group, which is also the largest one, is formed of hexagonal Te (space group P3121 (152), PDF 00–036-1452). There is no clear delimitation between the third and the fourth group, however above 60 at% of Te (Fig. 8d) only crystalline Te is found in the samples. In Fig. 8b,d, one can observe that the Ge and Te concentrations are positively correlated with the different crystalline phases. As we increase the Ge concentration, the crystalline phases formed are dominated by Ge-based phases and the reverse is true for Te. On the other hand, Te-rich compounds easily crystallize due to the isotropic atomic bonds^12^.

Si–Te phases have higher crystallization temperatures than Ge-Te, this is why Si–Te crystalline phases are never obtained in our samples (Fig. 8c). As shown by Koo et al.^59^, the SiTe composition crystallizes above 400 °C. Other crystalline Si–Te phases with a lower crystallization temperature are Si_2_Te_3_ and SiTe_4_. Si_2_Te_3_ crystalizes between 290 and 320 °C^2,60^, while SiTe_4_ at 236 °C^61^. There is more Ge than Si in the compositional library, except for only one sample with the highest amount of Si 45.6 at%, but a low amount of Te, 17.6 at%, which prohibits the formation of Si_2_Te_3_ or SiTe_4_. Tellurium starts to crystallize slightly above 100 °C^2^ while GeTe at 138 °C^12^. So, Te is always already consumed in other crystalline phases when the crystallization temperature of Si–Te phases is reached. Usually, in as deposited samples, Si spreads as a-Si or a-Ge_x_Si_1-x_ and delays the crystallization of Te or GeTe^2^ rather than forming Si–Te crystalline phases when Ge is also present in the material. Amorphous compositions above 400 °C, reported in a previous study^2^, had a Si concentration greater than 50 at%, which is not the case in our library.

## Conclusions

A Si–Ge–Te combinatorial thin films library was prepared using magnetron co-sputtering. The compositional, structural and optical properties were explored. The RBS measurements showed that an uninvestigated until now domain in the compositional space, was obtained: Si [7.7 ÷ 45.6] at%, Ge [11.2 ÷ 62.5] at%, Te [15 ÷ 69.8] at%. In the as-deposited state all the samples have amorphous structure and the order of the amorphous networks increases towards the GFD. The Tauc-Lorentz model used to fit the spectroscopic ellipsometry data allows for the determination of the thickness, bandgap, critical energies, optical dielectric constants and refractive indices in visible and near-IR. These properties are discussed in relation to the GFD of bulk glasses, the average coordination number and number of constraints. The bandgap varies between 0.71 eV and 1.02 eV and shows traces of a maximum at <*r*> ~ 2.75. The near-IR refractive index varies from 2.7 to 3.6, and has a minimum when the Ge concentration is between 28 and 45 at%. A threshold in the broadening parameter of the Lorentz oscillator is observed at 60 at% of Te, that suggests a transition to more ordered amorphous networks at the tie line between Ge_2_Te_3_ and Si_2_Te_3_. Two models, Lankhorst model and the stochastic agglomeration model, were used for the computation of the glass transition temperature. Annealing the library at 400 °C shows that all the samples crystallize, which confirms that the stochastic agglomeration model is more accurate in its predictions. Four groups of crystalline phases are identified which are positively correlated with the composition of the samples. Silicon does not take part in the formation of any crystalline phase up to 400 °C and remains in amorphous compounds. Its role is to delay or to prevent the crystallization of Te or GeTe. Above 60 at% Te, all the samples crystallize in hexagonal Te. These new findings in the Si–Ge–Te system enable a more informed and rapid decision making in material selection and design for applications in optical data storage, near-IR optical sensors and OTS devices.

## Methods

**Figure 9:**
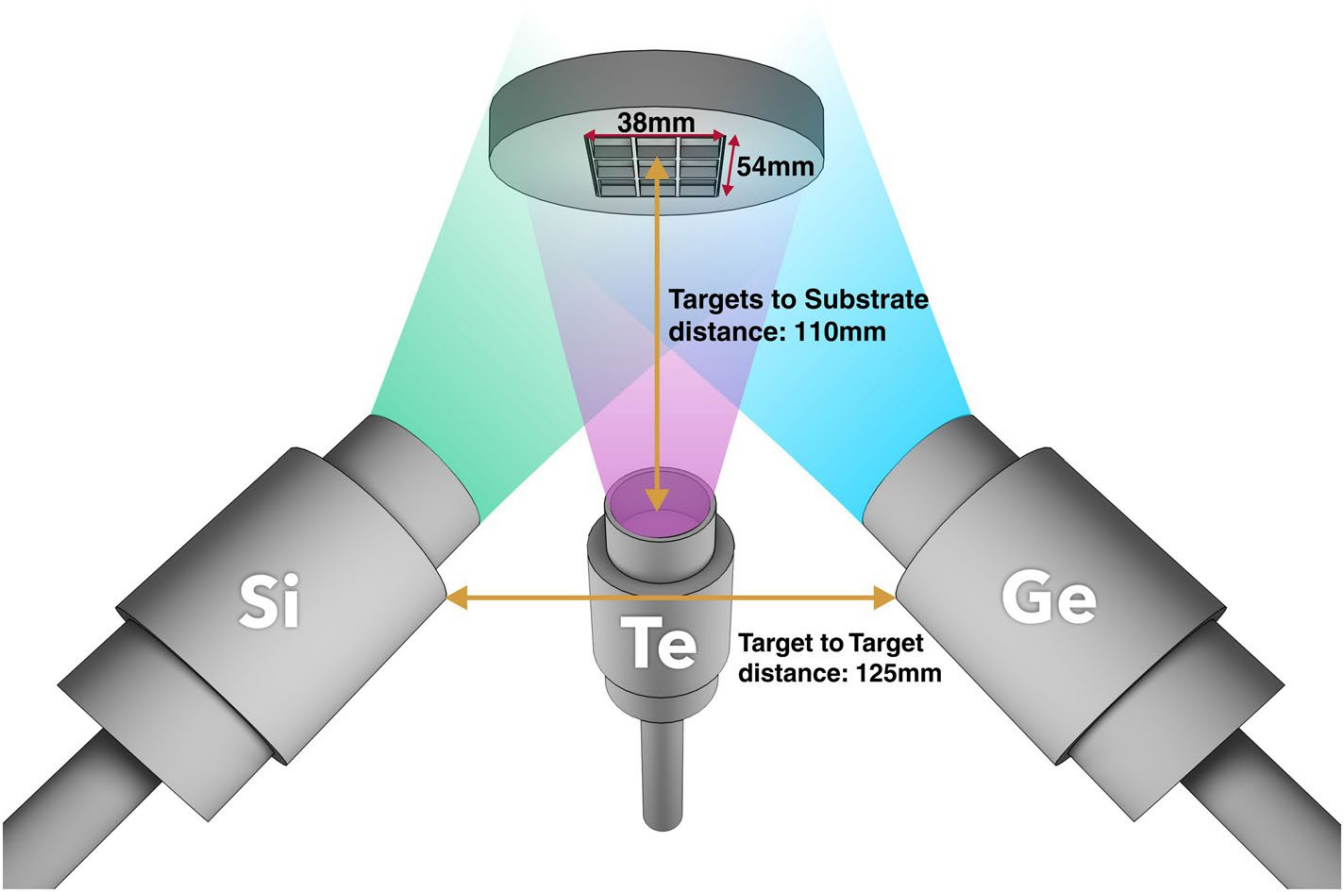
Schematic of the experimental combinatorial deposition setup. The three sputtering targets (Si, Ge and Te) are equidistant (125 mm) and placed at a 45 degree angle with respect to the substrate surface. The substrates are placed in the center of a circular holder above the targets at a distance of 110 mm. The figure was generated using 3ds Max 2020 (https://www.autodesk.com/) and CorelDRAW 11 (https://www.coreldraw.com/).

The magnetron sputtering system consists of a cylindrical deposition chamber with semi-spherical up and down caps. Six magnetrons are equidistantly placed on the bottom semisphere. Three of these six magnetrons were simultaneously used to sputter the Si, Ge and Te targets (99.99% purity, Mateck GmbH) and to obtain Si–Ge–Te combinatorial thin films. A set of substrates is placed on a holder in the upper part of the chamber. The distance between the central substrate and each magnetron is 11 cm (Fig. 9). Depositions with identical parameter settings were performed on graphite substrates for RBS measurements and on glass substrates for XRD and optical measurements. The deposition rates were optimized using an Inficon Q-bridge monitoring software connected to a quartz microcrystal. DC sputtering was used for the Ge and Si targets while RF sputtering was employed for the Te target. Powers between 10 and 50 W were applied on targets in order to obtain the targeted compositional range. Three series of depositions were performed and each time the deposition rate of Te was decreased in order to lower the Te concentration in the films. After initially evacuating the chamber to 10^–6^ Torr, Ar gas was introduced at a constant rate of 30 sccm, maintaining a stable pressure of 5 × 10^–3^ Torr during deposition. The surface of the targets was cleaned before deposition for 5 min with the shutters covering the magnetrons. The substrates were held at room temperature during depositions. In order to obtain thin layers suitable for structural, morphological and optical investigations, a thickness between 300 and 600 nm was pursued, resulting in a deposition time between 1500 and 2000s.

Rutherford backscattering spectrometry (RBS) experiments, using 3.041 MeV alpha particles delivered by a 3 MV Tandetron^62^, were performed in order to measure the samples composition. The measurements were made using a backscattering angle of 165°. The backscattered particles were registered with an AMETEK type BU-012-050-500 charged particle detector, having a solid angular acceptance of 1.641 msr, connected to a standard spectrometric chain and acquisition system. The typical energy resolution of the spectrometer was 18 keV. The RBS spectra were simulated using the SIMNRA software package^63^. The total combined standard uncertainty (accuracy) for each element is the following. For Si is 2.91% in the sample with the lowest Si concentration and 2.22% in the sample with the highest Si concentration, for Ge is 1.77% (lowest Ge concentration) and 1.26% (highest Ge concentration) and for Te is 1.37% (lowest Te concentration) and 1.14% (highest Te concentration), respectively.

The structure of the samples was investigated by X-ray diffraction at grazing incidence (GIXRD) using Cu K_α_ radiation (λ = 1.54178 Å) in a Rigaku SmartLab diffractometer, equipped with a HyPix-3000 2D Hybrid Pixel Array Detector (0D mode). The crystalline phases were identified using the DIFFRAC.EVA software. Only crystalline phases with a concentration above 5% were considered in the analysis.

Spectroscopic ellipsometry measurements were performed with a Woollam V-VASE system equipped with a HS-190 monochromator, at incidence angles of 50°, 60° or 70°, in the spectral range 0.7–5 eV. A priori, the glass substrates were measured in order to model their dielectric functions and to verify the quality and reproducibility of the surfaces. The backside reflections were suppressed by using a translucent adhesive tape^64^. The experimental results obtained were fitted using a substrate/thin film optical model. The WVASE32 software was used to evaluate the dielectric constants following the procedures described in ‘Guide to Using WVASE32′^65^. The complex dielectric function (*ε*) is related to the refractive index, *n*, and extinction coefficient, *k*, through the direct measurement of changes in polarization ψ and Δ.

The imaginary part of the dielectric function is fitted using a Tauc-Lorentz oscillator model, *ε*_2_ = (*E–E*_*g*_)^2^*/E* * *AE*_0_*Γ/[*(*E–E*_0_)^2^ + (*ΓE*)^2^]^50^, where *E*_*g*_ is the optical band gap, *E*_0_ is the resonance or critical energy, *A* is the amplitude and *Γ* is the broadening of the oscillator. The real part of the dielectric function, *ε*_1_, is related to *ε*_2_ by the Kramers–Kronig relation. The optical dielectric constant, *ε*_∞_, is the low energy-limit of *ε*_1_ and can be computed as ε_∞_ = ε_1_(0.05 eV)^66^. The electronic transitions are described by the resonance energy *E*_0_ of bound electrons oscillations, the broadening parameter *Γ* representing the scattering time of the carriers and the amplitude *A* given by the number of carriers making these transitions.

Annealing of the samples was performed in Ar atmosphere for 1 h at 400 °C using a MTI tubular furnace. We employ two models to estimate the glass transition temperature for the prepared library of compositions. The first model developed by Lankhorst^56^ computes *T*_*g*_ based on bond enthalpies for glasses predominantly covalently bonded, using an empirical relation, *T*_*g*_ = 3*.*44 * *H*_*a*_* − *480, where *H*_*a*_ is the enthalpy of atomization obtained by summing all individual bond enthalpies. In the second simulation, the Stochastic agglomeration model^57,58^ is used, which is based on the Gibbs-DiMarzio law^67^ and estimates the changes in the glass transition temperature with the chemical composition. *T*_*g*_ is computed as *T*_*g*_ = *T*_0_*/ln*(2) * *x* + *T*_0_, where *T*_0_ = *T*_*g*_ (*x* = 0). *T*_0_ was found by K. Gunasekera et al.^31^ to be 373 K, with *x* being the atomic concentration of Ge or Si.

## Supplementary Information

Supplementary Information.
