# Supplementary material for: Structural and optical properties of amorphous Si–Ge–Te thin films prepared by combinatorial sputtering
Source: Sci Rep. 2021 Jun 3;11:11755. doi: 10.1038/s41598-021-91138-x (PMC8175571; doi:10.1038/s41598-021-91138-x)
Supplement: Supplementary file 1 — Supplementary Information. [file 41598_2021_91138_MOESM1_ESM.pdf]

Supplementary Information

Structural and Optical Properties of Amorphous Si-Ge-Te Thin Films Prepared by Combinatorial Sputtering

C. Mihai[1], F. Sava[1], I. D. Simandan[1], A. C. Galca[1], I. Burducea[2], N. Becherescu[3], A. Velea[1, *]

[1]*National Institute of Materials Physics, Magurele, 077125, Romania*
[2]*Horia Hulubei National Institute of Physics & Nuclear Engineering, Magurele, 077125, Romania*
[3]*Apel Laser Ltd., Vanatorilor 25, Mogosoaia, 077135, Romania*

Corresponding Author
*E-mail: alin.velea@infim.ro

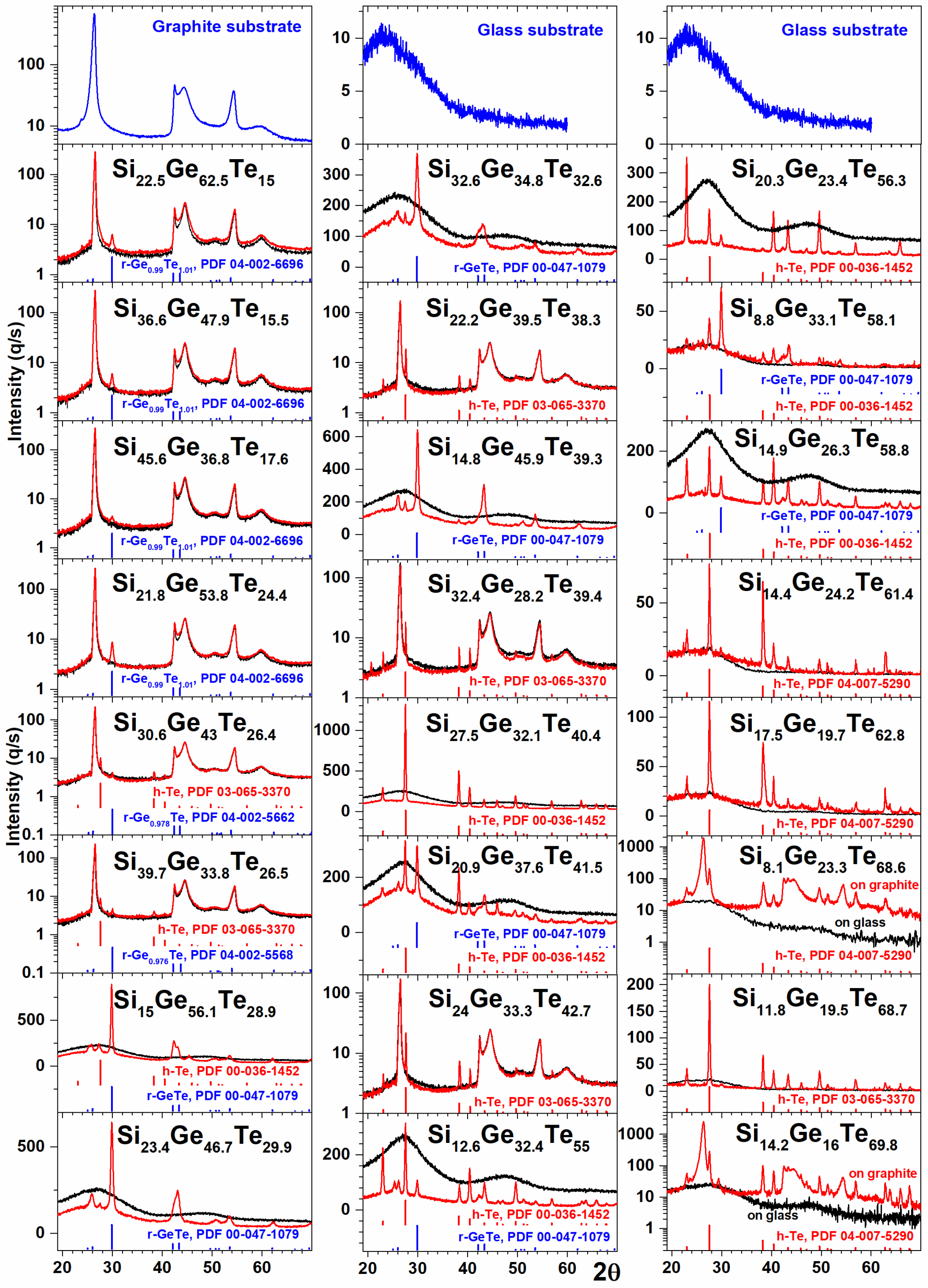

Figure S1. GIXRD diagrams of the Si-Ge-Te library: as-deposited (black curves) and annealed at 400 °C (red curves). The substrate of some samples is graphite, while for others is silicate glass.

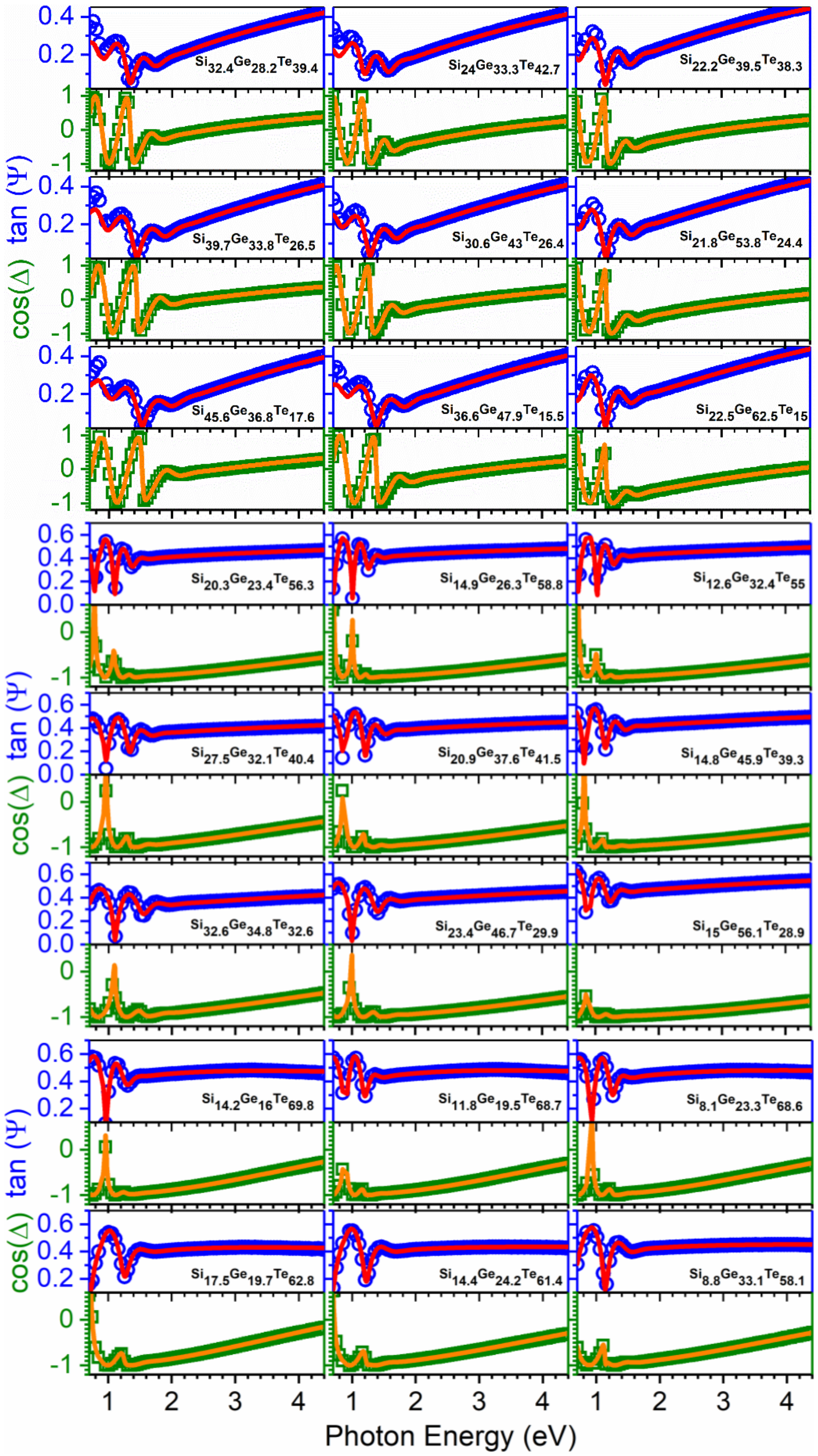

Figure S2. Spectroscopic ellipsometry results of the Si-Ge-Te library: Amplitude ratio upon reflection (tan(Ψ)) and phase shift (cos(Δ)). The experimental data is shown with blue and green dots whereas the model with red and orange lines.

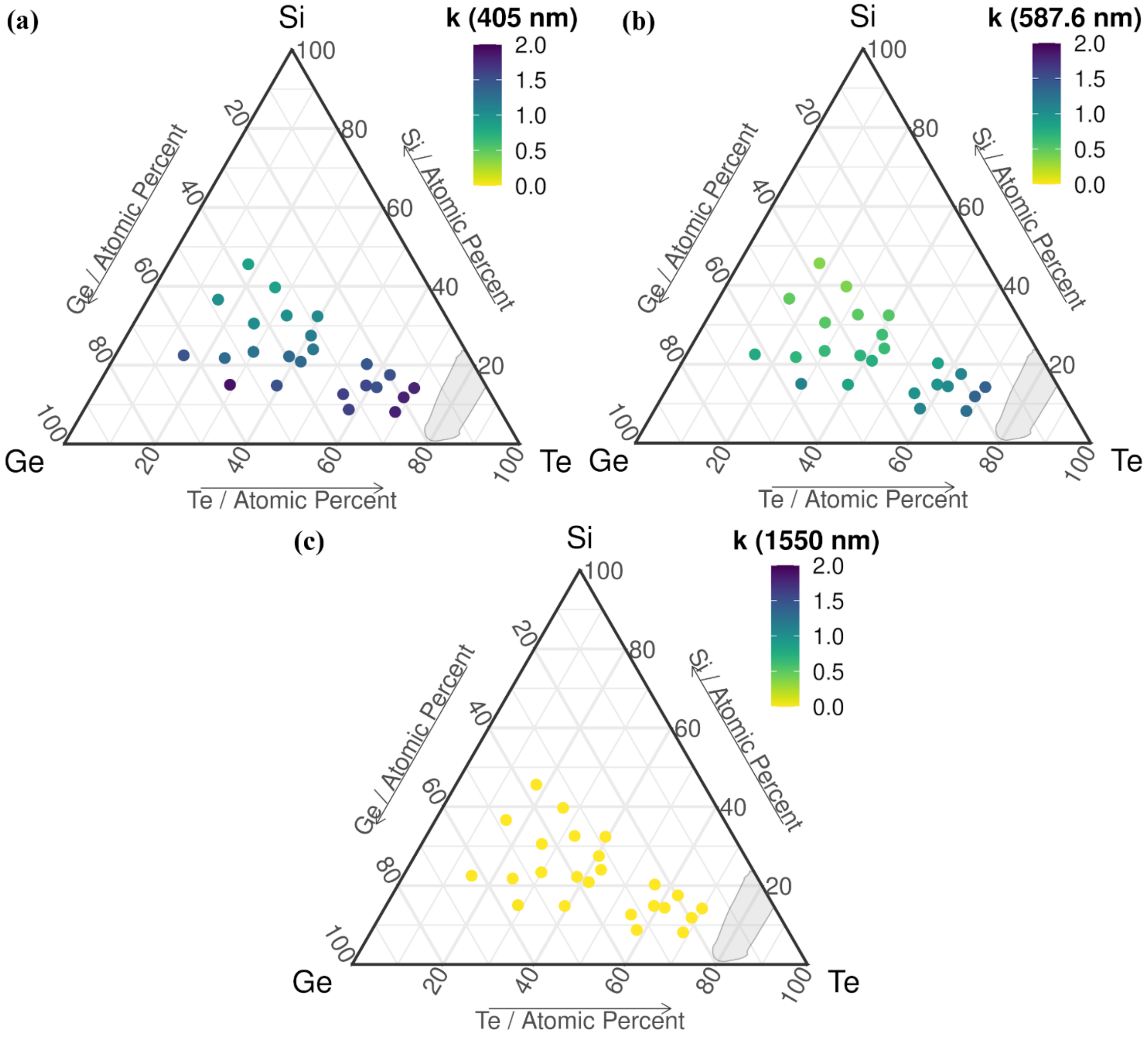

Figure S3. The extinction coefficient at different wavelengths: (a) 405 nm; (b) 587.6 nm and (c) 1550 nm.
